# Digital ischemic necrosis in a patient with systematic lupus erythematosus patient after severe acute respiratory syndrome coronavirus 2 infection: a case report

**DOI:** 10.1186/s13256-024-04567-3

**Published:** 2024-06-19

**Authors:** Jia-Hao Wen, Kai Zheng, Hua-Liang Ren, Wang-De Zhang, Chun-Min Li

**Affiliations:** Department of Vascular Surgery, Beijing Chaoyang Hospital, Capital Medical University, NO.8 Gongti South Road, Beijing, 100020 China

**Keywords:** Arterial thromboembolism, COVID-19, Systemic lupus erythematosus, Thrombosis

## Abstract

**Background:**

Patients with coronavirus disease 2019 have a high incidence of thrombosis that decreases after recovery. When coronavirus disease 2019 is accompanied by diseases prone to thrombosis, risk of post-infection thrombotic events may increase.

**Case presentation:**

We report a case of digital ischemic gangrene in a 24-year-old Chinese female with systemic lupus erythematosus after recovery from coronavirus disease 2019. The pathogenesis was related to clinical characteristics of systemic lupus erythematosus, hypercoagulability caused by coronavirus disease 2019, and second-hit due to viral infection.

**Conclusion:**

Patients with autoimmune diseases should remain alert to autoimmune system disorders induced by severe acute respiratory syndrome coronavirus 2 and other viruses. Treatment for these patients should be strictly standardized, and appropriate anticoagulation methods should be selected to prevent thrombosis.

## Introduction

Coronavirus disease 2019 (COVID-19) caused by severe acute respiratory syndrome coronavirus 2 (SARS-CoV-2) has a high thrombosis rate, although digital ischemic necrosis is relatively rare and mostly occurs in critically ill patients [1]. The incidence of thrombosis in patients after recovery from viral infection is only 2.5% [2]. This report describes a case of a young female patient with systemic lupus erythematosus (SLE) who suffered from digital ischemic gangrene after recovering from COVID-19.

## Clinical presentation

**Fig. 1:**
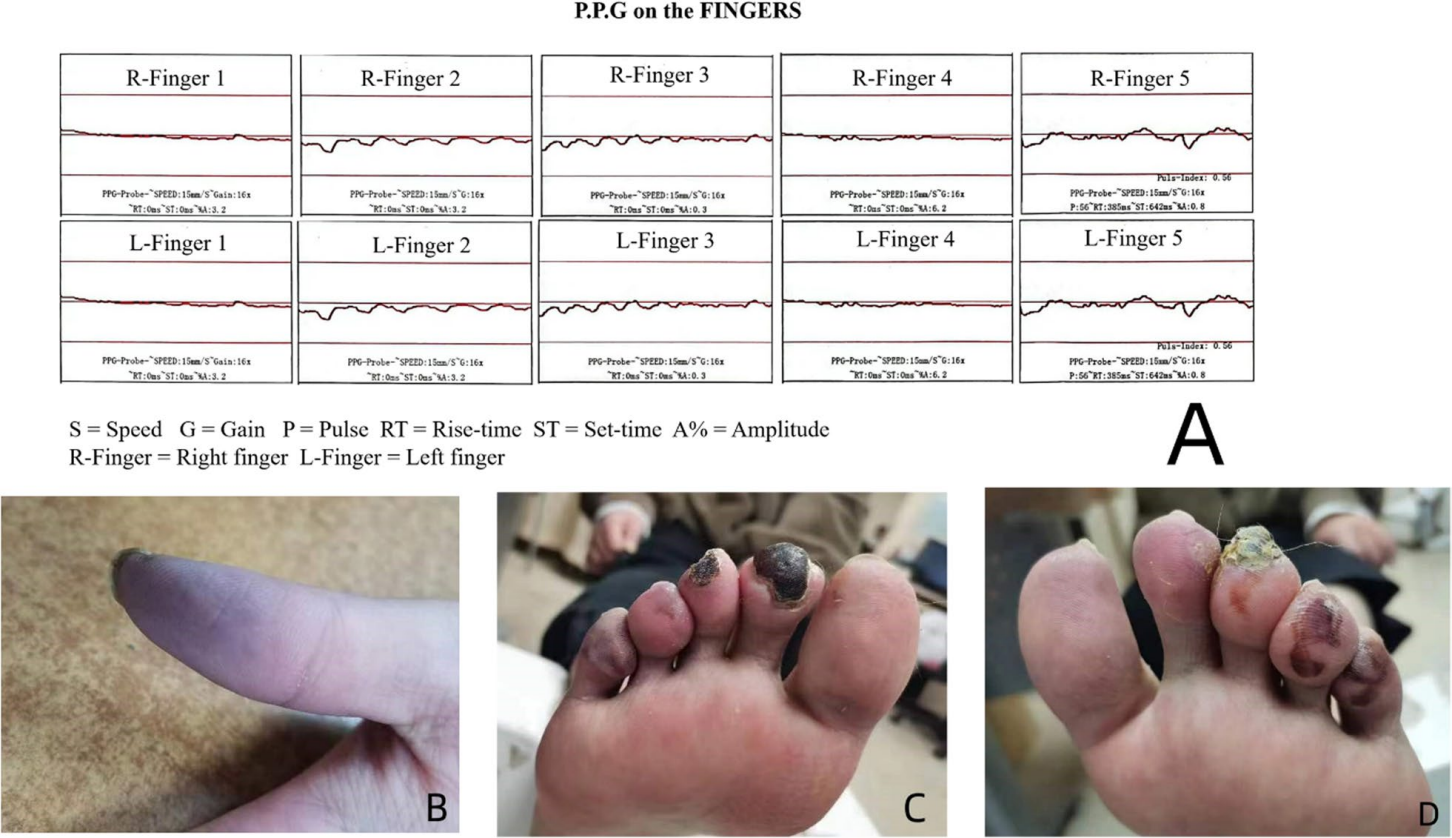
**A** The photoplethysmography measurement of finger artery perfusion showed a decrease in all finger artery amplitudes. **B**–**D** The right thumb was cyanotic, and the second toe of the right foot and the third toe of the left foot presented with ischemic necrosis

A 24-year-old Chinese female patient experienced gradual cyanosis in the hands and feet after recovering from COVID-19 4 months earlier. The end of the right little finger, the second toe of the right foot, and the third toe of the left foot presented with necrosis and were painful and numb. Erythema of the extremities was also present. The temperature of both hands and feet was low and the patient had cyanotic fingertips and black toes (Fig. 1). However, the bilateral radial, ulnar, posterior tibial, and dorsalis pedis arteries were pulsating well.

The patient was diagnosed with SLE and lupus nephritis 4 years prior and was treated with prednisone, cyclosporine, hydroxychloroquine, thalidomide, and benazepril. The patient voluntarily stopped using prednisone, hydroxychloroquine, cyclosporine, and thalidomide as symptoms improved 2 years earlier. Before the viral infection, the patient did not have any complaints.

The patient’s blood routine examination, liver and kidney function, and coagulation function showed no significant abnormalities. Routine blood test and coagulation function are presented in Table 1. Other abnormal laboratory tests are presented in Table 2. The patient’s SLE disease activity index (SLEDAI) score was 16, indicating severe activity. The photoplethysmography measurement of finger artery perfusion showed a decrease in all finger artery amplitudes, with the waveform appearing as a nearly horizontal straight line indicating peripheral circulatory disorders (Fig. 1).

**Table 1:** Routine blood test and coagulation function

Items	Results	Unit	Reference interval
WBC	5.41	×10^9^/L	3.5–9.5
NE	3.66	×10^9^/L	1.8–6.3
RBC	4.72	×10^12^/L	3.8–5.1
Hb	120	g/L	115–150
PLT	274	×10^9^/L	125–350
PT	12.4	Seconds	9.6–13.0
APTT	27.5	Seconds	23.3–32.5
INR	1.03		0.8–1.2
FBG	313.9	mg/dL	170.0–400.0
D-dimer	0.38	mg/L FEU	≤ 0.55

*WBC* white blood cell, *NE* neutrocyte, *RBC* red blood cell, *Hb* hemoglobin, *PLT* platelet, *PT* prothrombin time, *APTT* activated partial thromboplastin time, *INR* international normalized ratio, *FBG* fibrinogen

**Table 2:** Abnormal laboratory tests

Items	Results	Unit	Reference interval
Erythrocyte sedimentation rate	39	mm/hour	2–20
Urinary proteins	100	mg/dL	Negative
24-Hour urinary protein	781	mg	50–150
Urinary cast	5.9	/μL	0–0.9
Lupus anticoagulant	1.2		0.8–1.2
Anti-U1-snRNP antibodies	Positive (+)		Negative
Anti-SSB/La antibodies	Positive (+)		Negative
Anti-β2GP1 antibodies	28.23	RU/mL	< 20
Anti-cardiolipin antibodies	4.3	U/mL	< 10
Antinuclear antibodies	Positive		Negative
	S1:3200		< 1:100

The patient was diagnosed with SLE, lupus nephritis, antiphospholipid syndrome, and arterial thromboembolism.

Hydroxychloroquine, prednisone acetate, and mycophenolate mofetil were chosen to control the patient’s condition. Low-molecular-weight heparin was also administered, while sarpogrelate, kallidinogenase, nifedipine, papaverine, and beraprost sodium were given to improve microcirculation. The patient’s condition improved, with reduced cyanosis, improved limb circulation, and increased skin temperature. On a recent telephone follow-up, there was no further progress in the patient’s limb ischemia.

## Discussion

Endothelial injury, inflammation, and hypercoagulability are common clinical features of SLE [3]. Lupus anticoagulant, anti-cardiolipin antibodies, anti-β2-glycoprotein 1, and other autoantibodies are related to arterial and venous thrombosis. However, they increase the risk of thrombosis only under specific conditions, such as platelet activation [4]. Patients infected with SARS-CoV-2 can exhibit a series of coagulation abnormalities, such as thrombocytopenia, which serve as a prerequisite for thrombosis in patients with SLE [5]. At the same time, SARS-CoV-2 infection can induce autoimmune abnormalities and even trigger autoimmune diseases [6]. Therefore, viral infection can increase risk of lupus disease activity in patients with SLE, inducing vasculitis caused by strong SLE activity [4, 7]. Notably, the expression of angiotensin-converting enzyme 2 receptor increases in patients with SLE, providing more binding sites for SARS-CoV-2 [8]. The interaction between SLE and COVID-19 results in ischemic gangrene even in digital extremities with abundant blood circulation.

In addition to SARS-CoV-2 infection, severe SLE activity in patients is also related to their premature discontinuation of medication use. Hydroxychloroquinone is essential for the treatment of SLE, as it enhances the efficacy of mycophenolate mofetil in the treatment of lupus nephritis and has certain anti-thrombotic effects [9]. Aspirin and hydroxychloroquine are the primary preventive measures for patients with positive antiphospholipid antibodies, and long-term use of warfarin is necessary if the patient has experienced a thrombotic event [9].

For patients with SLE, it is also important to be vigilant about other viral infections since many viruses are related to SLE pathogenesis, especially Epstein–Barr virus, parvovirus B19, retroviruses, and cytomegalovirus [10]. There is also a significant correlation between seasonal influenza infection and SLE seizures [10]. There is significant peptide overlap among five common human viruses (influenza A, Borna disease, measles, mumps, and rubella) and human proteome [10]. Sequence similarity leads to an autoimmune response after viral infection, which can promote SLE activity and lead to adverse consequences.

## Conclusion

Although the peak of the COVID-19 pandemic has passed, autoimmune diseases either induced or exacerbated by SARS CoV-2 and other viruses should be alerted. Treatment for patients with SLE after infection with COVID-19 or other viruses should be strictly standardized and appropriate anticoagulation methods should be selected to prevent thrombosis.

## Data Availability

Data of the patient can be requested from the authors.
